# Association of Anxiety and Depressive Symptoms and Treatment Adherence Among Patients With Type 2 Diabetes Mellitus in Jazan, Saudi Arabia: A Cross-Sectional Study

**DOI:** 10.7759/cureus.56068

**Published:** 2024-03-13

**Authors:** Suhaila A Ali, Sarah M Salih, Amani Abdelmola, Anwar M Makeen, Yahia M Solan, Mona M Mohammed Ali

**Affiliations:** 1 Department of Family and Community Medicine, Faculty of Medicine, Jazan University, Jazan, SAU; 2 Department of Family Medicine, Jazan Diabetes and Endocrinology Center, Jazan, SAU; 3 Department of Pediatric Endocrinology, Jazan Diabetes and Endocrinology Center, Jazan, SAU

**Keywords:** type 2 diabetes mellitus, kingdom of saudi arabia (ksa), jazan, anxiety, depression, patient health questionnaire-4 (phq-4), adherence, diabetes type 2

## Abstract

Background: Diabetes mellitus is a serious public health concern. It is associated with many psychological problems, such as depression, anxiety, and eating disorders. These co-morbidities are associated with improper adherence to treatment, self-care, poor glycemic control, more complications, and worse outcomes.

Methods: This study aimed to measure the level of medication adherence among type 2 diabetics in Jazan, Saudi Arabia, and to find its association with their psychological status (specifically, depression and anxiety). A cross-sectional descriptive design was used among adults with type 2 diabetes at the Diabetes and Endocrinology Center in Jazan, Saudi Arabia. The estimated sample size was 480 patients. The General Medication Adherence Scale and Patient Health Questionnaire-4 (PHQ-4) were used as tools to achieve the study objectives.

Results: A total of 449 diabetic patients completed the survey (93.5% response rate). Patients with poor, low, and partial adherence account for 337 (75%) of patients and only 112 (25%) have good and high medication adherence. Employment and duration of illness were highly significant with a positive relationship to treatment adherence (p = 0.010 and 0.000, respectively). On the other hand, age and disease duration had a significant relationship with psychological disorders (p = 0.029 and 0.002, respectively). Of the patients, 64 (14.3%) had high scores on the PHQ-4, with depressive symptoms in 46 (10.24%) and anxiety symptoms in 75 (16.7%). Correlation analysis reveals that there is a highly significant negative correlation between psychological disorders and adherence to medications (r = -0.288, p = 0.000).

Conclusion: A negative correlation between psychological disorders and adherence to medications was found. The findings indicate the importance of psychological support for diabetic patients for better treatment adherence.

## Introduction

Diabetes mellitus is a serious chronic metabolic condition that is increasing steadily worldwide [1,2]. It is characterized by hyperglycemia due to insufficient insulin or insulin resistance. The total number of diabetics was 537 million in 2021 [2], and there is an estimated increase by the year 2045 ranging from 13% in Europe to 138% in African regions. An 87% increase is expected in the Middle-Eastern region [2]. More than 95% of people with diabetes have type 2 diabetes, while type 1, gestational, and latent types account for the remaining 5% [1].

The prevalence in Saudi Arabia is about 18.7% [2], making it among the highest in Gulf countries where the prevalence ranges from 8% to 22% [3]. According to the International Diabetes Federation, one in every six Saudis is diabetic and yet 43.6% are still not diagnosed in the age group 20 to 79 years [2].

Diabetes is a public health concern because of the many complications it causes and the burden it has on health systems, families, and the patients themselves. Complications include diabetic retinopathy, neuropathy, kidney failure, and cardiovascular disease [1]. Diabetes is also associated with serious co-morbidities like psychiatric and psychological disorders (depression, anxiety, and eating disorders) [4-7].

Studies show that the risk for depression is twice as high or more among diabetic patients and that stress is even higher [5,7]. The gravity of this double morbidity is that it is associated with improper adherence to treatment, self-care, poor glycemic control, more complications, and worse outcomes [4-8].

Treatment adherence according to the World Health Organization is the degree to which a person’s medicine-taking behavior, dietary habits, and/or implementation of lifestyle changes relate to agreed recommendations from his/her healthcare provider [9]. Studies on diabetic patients show adherence ranged from 38.5% to 93.1% [10]. Many factors are found associated with poor adherence like drugs not tolerated by the patient, more doses of treatment, higher cost, presence of depression and other psychological morbidities, and forgetfulness [10-12].

Studies show that addressing psychosocial issues results in improved adherence, better glycemic control, fewer hospital visits and hospitalizations, and decreased medical costs [10-13].

In Saudi Arabia, many studies show poor drug adherence in more than 40% of diabetic patients [8]. Moreover, psychological co-morbidities were noted. Studies in the southern province of Saudi Arabia found approximately 9.6% to 15% of diabetic patients have major depression [6,14], compared to 9% of the general population [6]. The prevalence of diabetes in Jazan was around 12% in 2015 [15].

Therefore, this study aimed to measure the level of medication adherence among type 2 diabetes mellitus (T2DM) patients in Jazan, Saudi Arabia, and to find its association with their anxiety and depressive symptoms.

## Materials and methods

Study design

A cross-sectional descriptive design was used to determine treatment adherence among type 2 diabetics in Jazan, Saudi Arabia, and its association with their psychological status.

The study was conducted at the Diabetes and Endocrinology Center in Jazan, Saudi Arabia, among T2DM patients. The center is a referral facility for diabetic patients from all primary healthcare centers and hospitals in the Jazan region serving about 10,000 diabetic patients [6].

Included in the study are adult patients (both male and female, aged 18 years or older) with T2DM, attending the Diabetes and Endocrinology Center in Jazan, and having a medical file in the center. Moreover, those capable of independent communication and who gave informed written consent were included. Patients with other types of diabetes were excluded from the study.

The calculated sample size is 480 diabetic patients from the total number of diabetic patients in the Jazan area, calculated using the formula for cross-sectional studies with a 95% confidence interval and 5% margin of error, and a further 20% increase to account for non-response.

Subjects were recruited by daily total coverage of the attending patients to the center who met the inclusion criteria. The recruitment of subjects and taking their weight and height were done by trained nurses at the center.

Methods and tools

The study used a structured, self-administered, validated questionnaire in Arabic that included sociodemographic information, diabetes history, the Arabic version of the Patient Health Questionnaire-4 (PHQ-4) [16], and the General Medication Adherence Scale (GMAS) [8].

The PHQ-4 is a screening test translated and validated in Arabic and adapted to the Saudi population by Alhadi et al. [16]. It consists of the first two items of the PHQ-9 and the General Anxiety Disorder-7 (GAD-7). The PHQ-4 is the combination of the two core items for major depressive disorder and generalized anxiety disorder in the Diagnostic and Statistical Manual of Mental Disorders, Fourth Edition (DSM-IV). The scores range from 0 to 12, and the cutoff point for each sub-scale is 3. The higher the score, the more likely there is an underlying depressive or anxiety disorder. It is a proven measure for depression and anxiety screening.

The GMAS is an 11-item self-reporting medication adherence measurement that was developed originally in Urdu and translated and validated in Arabic for Saudi patients by Naqvi et al. [8]. The total score is 33. Patient scores were interpreted as high adherence (30-33), good (27-29), partial (17-26), low (11-16), and poor (≤10).

Data management

SPSS version 24 (IBM Corp., Armonk, NY) was used to find the significant relations, with a confidence interval of 95% and p-value of 0.05. Descriptive statistics, linear regression, and correlation tests were used to analyze the collected data.

A pilot study was conducted on 20 subjects with consideration of inclusion and exclusion criteria to assure the quality of data, test the questionnaire's clarity, and avoid design issues. Results are not included in the final data analysis of the study.

Ethical consideration

This research was conducted according to ethical standards and was approved by the Jazan Ethics Committee (national registration number: NCBE-KACST, KSA: H-10-Z-073) with approval number 2057.

Informed written consent with a waiver of identity was obtained from all participants. All data were kept confidential and only used for the research.

## Results

Demographic information

A total of 449 diabetic patients completed the study (93.5% response rate). Females represent 232 (51.7%) and males represent 213 (47.4%) patients. Most participants were Saudi nationals (412, 91.8%), in the age group of 36 to 55 years (195, 43.4%), married (260, 57.9%), with a university degree (183, 40.8%), employed (229, 51%), and with a monthly income less than 5000 Saudi riyals (about 1333 United States dollars) (218, 48.6%). Regarding patients' weight, about 32% were equally normal weight (145), overweight (144), and obese (146), while only five (1.1%) were underweight. The complete details of the demographic information are shown in Table 1.

**Table 1:** Demographic characteristics, history of type 2 diabetes, and co-morbidities among the studied population.

Factor	Frequency	Percent
Gender		
Male	213	47.4
Female	232	51.7
Did not disclose	4	0.9
Total	449	100
Social status		
Single	79	17.6
Married	260	57.9
Divorced	52	11.6
Widow	49	10.9
Did not disclose	9	2
Total	449	100
Age group		
19-35	140	31.2
36-55	195	43.4
More than 55	114	25.4
Total	449	100
Educational level		
Uneducated	56	12.4
Primary	41	9.1
Intermediate	57	12.7
Higher secondary	108	24.1
University	183	40.8
Did not disclose	4	0.9
Total	449	100
Nationality		
Saudi	412	91.8
Non-Saudi	33	7.3
Did not disclose	4	0.9
Total	449	100
Employment		
Employed	229	51.0
Unemployed	210	46.8
Did not disclose	10	0.2
Total	449	100
Monthly income in Saudi Riyal		
Less than 5,000	218	48.6
5,000-10,000	34	7.6
More than 10,000	136	30.3
Did not disclose	61	13.5
Total	449	100
BMI		
Underweight	5	1.1
Healthy weight	145	32.3
Overweight	144	32.1
Obese	146	32.5
Weight not measured	9	2
Total	449	100
Duration of diabetes		
Less than a year	113	25.2
1-5 years	99	22.0
6-10 years	86	19.2
More than 10 years	148	33.0
Did not disclose	3	0.6
Total	449	100
Is someone taking care of your medication?		
Yes	198	44.1
No	103	22.9
Sometimes	142	31.6
Did not disclose	6	1.3
Total	449	100
Other co-morbidities		
Asthma	11	2.4
Blindness	1	0.2
Blood pressure	101	22.5
Heart disease	14	3.1
Kidney disease	1	0.2
Mental and psychological	1	0.2
Others	50	11.3
No disease	270	60.1
Total	449	100

The medical history of the respondents showed that 113 (25%) were diagnosed with T2DM for less than a year and 148 (33%) for more than 10 years. A total of 198 (44%) had someone taking care of their medication.

A total of 40% had other co-morbidities with diabetes; 101 (22.5%) were hypertensive, 14 (3.1%) had heart disease, one (0.2%) had kidney disease, and only one patient (0.2%) reported mental illness. The complete details of the sample’s medical history are shown in Table 1.

Medication adherence

Patients with poor, low, and partial adherence accounted for 337 (75%) of patients, with 50 (11.1%), 110 (24.5%), and 177 (39.4%) patients, respectively. Only 112 (25%) patients had good and high medication adherence. The detailed scores of the GMAS are shown in Table 2.

**Table 2:** Medication adherence scores of T2DM patients using GMAS. T2DM: type 2 diabetes mellitus; GMAS: General Medication Adherence Scale.

General Medication Adherence Score (total score = 33)	Frequency	Percentage
Poor adherence (≤10)	50	11.1
Low adherence (11-16)	110	24.5
Partial adherence (17-26)	177	39.4
Good adherence (27-29)	60	13.4
High adherence (30-33)	52	11.6
Total	449	100

Demographic factors and medication adherence

Results of linear regression analysis show that the relationship between demographic factors and adherence to medications is not significant, except in the case of employment and duration of illness, which indicated a highly significant and positive relationship to treatment adherence (0.010 and 0.000, respectively). Detailed results are presented in Table 3.

**Table 3:** Regression analysis of demographic factors and medication adherence. ^a^ Dependent variable: adherence to medications.

Coefficients^a^						
Model		Unstandardized coefficients		Standardized coefficients	t	Sig.
		B	Std. error	Beta		
1	Constant	20.625	4.391		4.697	0.000
	Gender	1.260	0.819	0.082	1.538	0.125
	Social status	-0.710	0.517	-0.077	-1.373	0.171
	Age group	-0.412	0.607	-0.040	-0.678	0.498
	Education Level	0.649	0.407	0.115	1.594	0.112
	Nationality	-0.597	1.744	-0.017	-0.342	0.732
	Employment	2.715	1.047	0.174	2.594	0.010
	Monthly income	0.218	0.528	0.026	0.412	0.681
	BMI	-0.396	0.455	-0.045	-0.870	0.385
	How long have you been diabetic?	2.527	0.379	0.392	6.671	0.000
	Is there someone taking care of your medication?	0.059	0.475	0.007	0.124	0.902

Prevalence of mental symptoms

**Figure 1:**
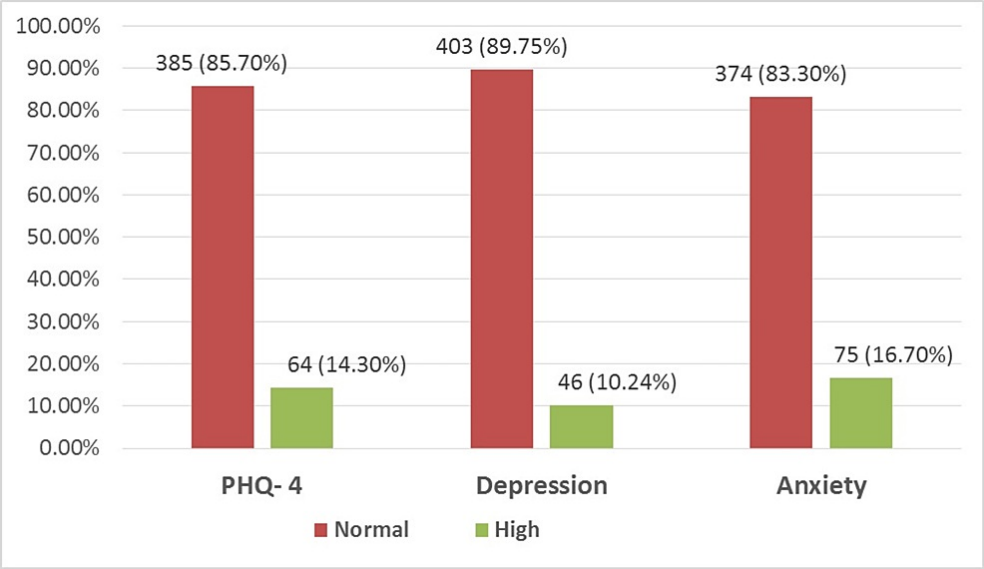
Prevalence of mental symptoms among diabetic patients using the PHQ-4 scale. PHQ-4 score: Normal (0-6) and high (7-12). Depression sub-scale score: Normal (0-3) and high (4-6). Anxiety sub-scale score: Normal (0-3) and high (4-6). PHQ-4: Patient Health Questionnaire-4.

More than 64 (14%) of patients have high scores on the PHQ-4 measurement, indicating the presence of an underlying depressive or anxiety disorder. Depressive symptoms were found in 46 (10.24%) and anxiety symptoms were found in 75 (16.7%), while 46 patients had symptoms of both depression and anxiety (10.24%). Most of the respondents (166, 37.0%) have a lack of interest and pleasure when doing things more than half of the days. Moreover, most patients (276, 61.5%) were feeling uncomfortable, depressed, or hopeless some days of the week. Another 265 (59.0%) were feeling stressed, nervous, and anxious for some days of the week, and most of them (148, 33.0%) could not control anxiety and worries for more than half of the days. Details of psychological disorder scores are shown in Figure 1.

Demographic factors and psychological disorders

The linear regression analysis has been carried out to define the relationship between demographic factors and psychological disorders. The first was taken as an independent variable and the second as a dependent variable. Results indicated that only age and disease duration have a significant relationship with psychological disorders (0.029 and 0.002, respectively). Age has a positive association while disease duration has a negative association with psychological disorders, as presented in Table 4.

**Table 4:** Regression analysis of demographic factors and psychological disorders. ^a^ Dependent variable: psychological disorders.

Coefficients^a^						
Model		Unstandardized coefficients		Standardized coefficients	t	Sig.
		B	Std. error	Beta		
1	Constant	7.252	1.593		4.552	0.000
	Gender	0.358	0.293	0.068	1.220	0.223
	Social status	0.204	0.180	0.065	1.133	0.258
	Age group	0.471	0.215	0.132	2.195	0.029
	Education	-0.025	0.146	-0.013	-0.175	0.861
	Nationality	0.717	0.639	0.058	1.122	0.263
	Employment	-0.662	0.376	-0.123	-1.759	0.079
	Monthly income	-0.182	0.188	-0.064	-0.964	0.336
	Body mass index	-0.052	0.163	-0.017	-0.320	0.749
	Duration of diabetes	-0.413	0.134	-0.188	-3.085	0.002
	Someone taking care of the patient's medication	0.279	0.170	0.093	1.637	0.103

The association between psychological disorders and treatment adherence

Correlation analysis reveals that there is a highly significant negative correlation between psychological disorders and adherence to medications (r = -0.288, p = 0.000). The results of the correlation analysis are shown in Table 5.

**Table 5:** Correlation analysis of treatment adherence and psychological disorders. ** Correlation is significant at the 0.01 level (two-tailed).

Correlations			
		Psychological disorder	Adherence to medications
Psychological disorder	Pearson correlation	1	-0.288^**^
	Sig. (2-tailed)		0.000
	N	444	410
Adherence to medications	Pearson correlation	-0.288^**^	1
	Sig. (2-tailed)	0.000	
	N	410	415

## Discussion

This study aimed to measure the level of medication adherence among type 2 diabetics and to find its association with their psychological status (depression and anxiety). The study provided valuable evidence on the improper level of treatment adherence among the studied population. It also highlighted that age had a positive association with the presence of a psychological disturbance while diabetes mellitus duration had a negative association. Of the patients, 64 (14.3%) had high scores on the PHQ-4, indicating the presence of depression and anxiety symptoms. Correlation analysis revealed that there is a highly significant negative correlation between psychological disorders and adherence to medications.

The majority of the sample (337, 75%) had improper treatment adherence; poor adherence among 50 (11%), low among 110 (24.5%), and partial adherence among 177 (39.4%). Only 112 (25%) had good and high adherence scores on GMAS. These findings contrast with other studies in the Kingdom of Saudi Arabia (KSA) that found higher treatment adherence ranging from 33% to 77% among studied populations [13,17-19]. However, only one of these studies in Khobar, KSA used the same GMAS scale [17]. The main difference between this study and the one that used the GMAS is that the majority of the patients in this study were from a low socioeconomic class (48.6% with less than Saudi riyal (SAR) 5000 monthly income), while in the other study, 53.8% had a monthly income above SAR 10,000 [17]. Although the socioeconomic class had no statistically significant association with treatment adherence, this factor might need to be studied further. Only employment status and duration of disease had a statistically positive association with treatment adherence.

Similar low adherence levels were observed in 54.8% of the participants in a tertiary hospital in Riyadh, KSA [20], 76.9% in a multi-center study in Ethiopia [21], and 66.5% in a hospital-based study in Ghana [22].

Regarding the psychological status of the studied sample, depressive symptoms were found in 46 (10.24%), and anxiety symptoms were found in 75 (16.7%), with overall 64 (14.3%) of the participants showing signs of underlying psychological disturbance. What is alarming is that only one patient (0.2%) of the sample reported the presence of a psychological problem, this could indicate that many of these patients are unaware of their psychological status.

These findings are consistent with the findings of other studies in KSA and internationally. A previous study in the Jazan region found the prevalence of moderate and severe depression among T2DM patients to be 13.8% [14]. A recent study found depression at 54.4% and anxiety at 47.1% [6]; however, this study was conducted during the COVID-19 pandemic, which might explain this high percentage compared to our recent study.

A study in western KSA found the prevalence of depression and anxiety at 33.8% and 38.3%, respectively [23]. A study exploring the health-related quality of life among diabetic patients in three major cities in KSA found a prevalence of 45.8% of anxiety and depression using the same PHQ-4 tool as in this study [24]. In China, a study found the incidence of depression was approximately 24% [7]. In a hospital-based study in Pakistan, 50.7% of patients had anxiety and 49.2% had depression [25].

A large systematic review has found a global average of 28% depression among T2DM patients, and the rate is higher in Asia reaching 32% [26]. The prevalence of anxiety was found at 18% in a study conducted among T2DM patients in 15 countries [27]. These findings are much higher than what this study reported, this might be due to the different tools used or the difference in populations, since some studies were hospital-based and others were outpatient-based. Moreover, some were conducted during the COVID-19 pandemic. Moreover, many different social factors exist. A proper psychological evaluation needs to be conducted to verify the findings of this study.

Factors associated with psychological disorders in this study are age and disease duration. Age had a positive association with the presence of a psychological disturbance while diabetes mellitus duration had a negative association. No association was found with sex, education level, or employment. The results of other studies in the Jazan region were also varied. One study found no association with age, sex, or duration of diabetes [14], while another found depression was higher in females, young age, and unemployed participants [6]. Another study in western KSA found age has a negative correlation with depression [23]. A large meta-analysis study found that depression was more common in females (34% vs. 23% in males) and those younger than 65 years old [26].

Notably, this study found a highly significant negative correlation between treatment adherence and psychological disorders. These findings corroborate with other reliable scientific evidence across countries and use different methodologies that show the negative effects of psychological disorders (depression and anxiety) on treatment adherence among diabetic patients [28,29].

Depression and diabetes distress were found to negatively affect treatment adherence in studies in China [7] and Ghana [22]. Patients with depression and anxiety were found to be more nonadherent than those with no cognitive impairment in a study in outpatient clinics in Portugal [30]. This association was also found in a study in western KSA and in a review of 36 studies [23,29].

Depression and anxiety were also acknowledged as predictors and modifiable factors for treatment adherence [10,30]. The evidence of the association between psychological disorders and non-adherence to medication among T2DM necessitates that health authorities and decision-makers take this into account when tackling the issue of treatment adherence, patient well-being, and quality of life among T2DM patients.

Limitations

The study has some limitations, including the descriptive nature of the study that provides associations and no causality can be established. The psychological status of the patients was determined using screening tools and not a thorough psychological assessment. Another limitation is the possibility of recall bias among the sample, and that it was conducted in one center. These limitations make the generalizability of the results to other populations and geographical regions impossible.

## Conclusions

This study highlights the high percentage of improper treatment adherence among T2DM patients in Jazan, Saudi Arabia, and the evidence of psychological disturbance among some of these patients. Factors to consider in relation to treatment adherence are employment status and duration of illness; both have a significant positive relation with treatment adherence. The study also provides evidence of psychological disturbance among type 2 diabetics and the negative correlation between psychological disorders, and improper treatment adherence was found to be highly significant. While age has a positive association with psychological disorders, T2DM disease duration has a negative one.

These findings indicate the importance of psychological support for diabetic patients and that any management plans need to consider psychological support for better treatment adherence. Regular screening at primary healthcare centers or diabetes centers should be considered, especially since screening tools are widely available and have proven effective in detecting high-risk cases. Further studies using more advanced techniques are recommended to establish cause-and-effect relationships between treatment adherence and the psychological status of diabetic patients.
